# El Niño Impact on Mollusk Biomineralization–Implications for Trace Element Proxy Reconstructions and the Paleo-Archeological Record

**DOI:** 10.1371/journal.pone.0054274

**Published:** 2013-02-06

**Authors:** Alberto Pérez-Huerta, Miguel F. Etayo-Cadavid, C. Fred T. Andrus, Teresa E. Jeffries, Clifton Watkins, Shane C. Street, Daniel H. Sandweiss

**Affiliations:** 1 Department of Geological Sciences, The University of Alabama, Tuscaloosa, Alabama, United States of America; 2 Mineralogy Department, Natural History Museum, London, United Kingdom; 3 Department of Chemistry, The University of Alabama, Tuscaloosa, Alabama, United States of America; 4 Climate Change Institute/Department of Anthropology, University of Maine, Orono, Maine, United States of America; National Institute of Water & Atmospheric Research, New Zealand

## Abstract

Marine macroinvertebrates are ideal sentinel organisms to monitor rapid environmental changes associated with climatic phenomena. These organisms build up protective exoskeletons incrementally by biologically-controlled mineralization, which is deeply rooted in long-term evolutionary processes. Recent studies relating potential rapid environmental fluctuations to climate change, such as ocean acidification, suggest modifications on carbonate biominerals of marine invertebrates. However, the influence of known, and recurrent, climatic events on these biological processes during active mineralization is still insufficiently understood. Analysis of Peruvian cockles from the 1982–83 large magnitude El Niño event shows significant alterations of the chemico-structure of carbonate biominerals. Here, we show that bivalves modify the main biomineralization mechanism during the event to continue shell secretion. As a result, magnesium content increases to stabilize amorphous calcium carbonate (ACC), inducing a rise in Mg/Ca unrelated to the associated increase in sea-surface temperature. Analysis of variations in Sr/Ca also suggests that this proxy should not be used in these bivalves to detect the temperature anomaly, while Ba/Ca peaks are recorded in shells in response to an increase in productivity, or dissolved barium in seawater, after the event. Presented data contribute to a better understanding of the effects of abrupt climate change on shell biomineralization, while also offering an alternative view of bivalve elemental proxy reconstructions. Furthermore, biomineralization changes in mollusk shells can be used as a novel potential proxy to provide a more nuanced historical record of El Niño and similar rapid environmental change events.

## Introduction

Biological consequences of El Niño events are well documented [1], with a clear disruption of fisheries and mass mortality of bivalve mollusks in the coastal areas of Peru and Ecuador [1], [2]. Some bivalve and gastropod species, however, survive and continue the production of shell components during these events. These surviving mollusk shells, therefore, represent ideal proxy-bearers for reconstructions of climatic and environmental changes associated with El Niño along the west coast of South America [3], [4]. Many fossil shell accumulations are found in Pleistocene and Holocene coastal deposits as well as archeological sites [2]–[4], potentially extending reconstructions for past events. Isotope and radiocarbon data from bivalve shells have been used to characterize anomalies in sea surface temperature and upwelling triggered by El Niño [3], [5]–[7]. In contrast, trace element proxies (metal-to-calcium ratios [Me/Ca]) of bivalve shells have been scarcely explored for the characterization of El Niño events [4], [8], despite their usefulness in climatic and environmental studies [9]–[11]. Prior to the complete exploitation of these elemental proxies, the environmental impact on biomineralization processes induced by El Niño has to be assessed. Preliminary studies suggest that surviving bivalves experience levels of environmental stress responsible for morphological changes in shells [2], [3].

Here, we analyze biomineralization structural changes and trace element variations in specimens of *Trachycardium procerum*, a large infaunal cardioidean bivalve, collected from the Peruvian coast after the 1982–83 El Niño [2]–[4].

## Materials and Methods

### Samples

**Figure 1:**
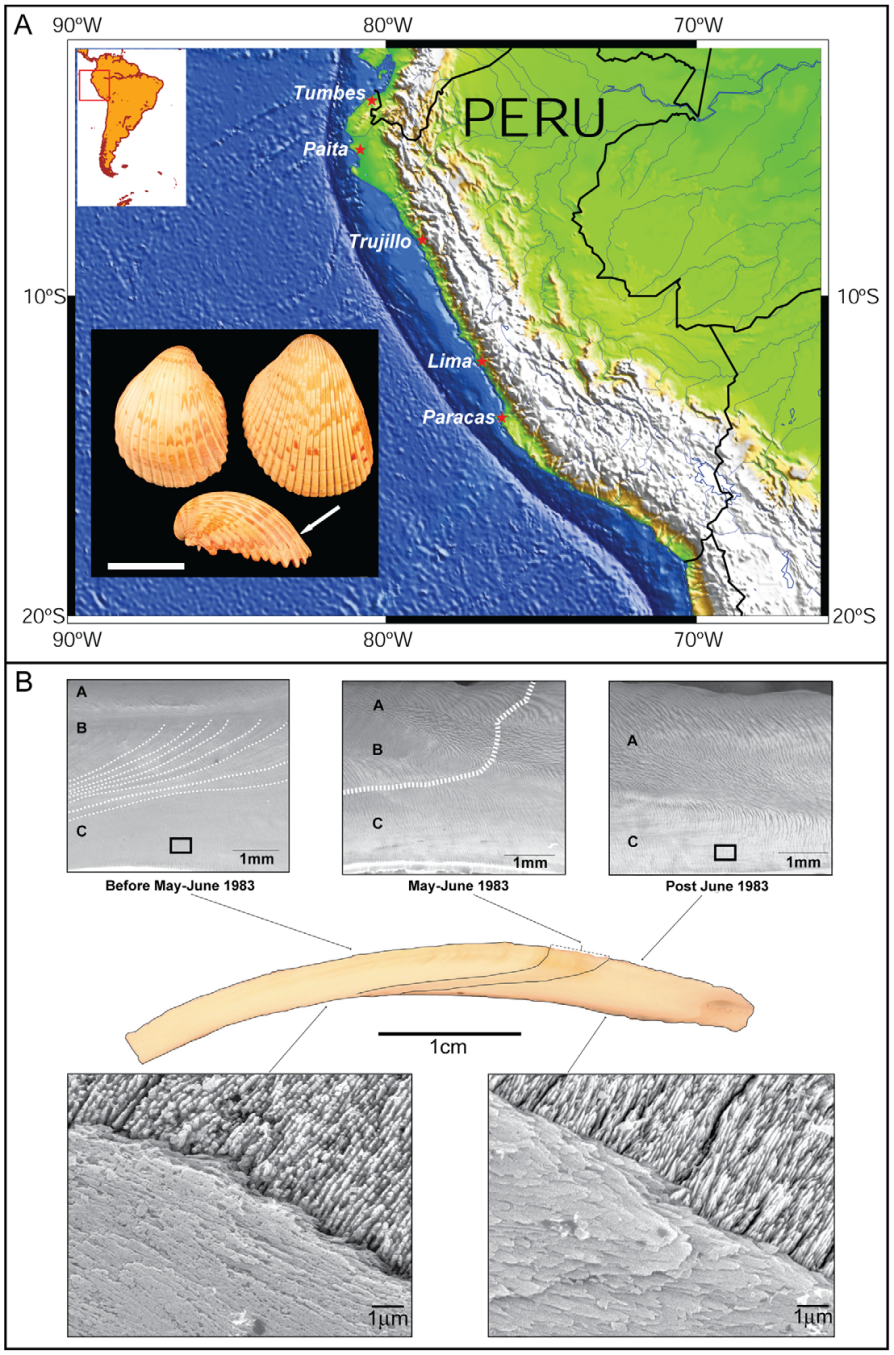
Map with collection site of *T. procerum* specimens and shell microstructures related to 1982–1983 El Niño event. (A) Shell collection site at Paracas in the coast of Peru and an image of *T. procerum* shells (insert) [white arrow indicates the location of the scar; scale = 4 cm]. (B) SEM images of shell regions in specimen 2TP4-2 precipitated during (before May–June 1983; A, B and C layers, with growth lines indicated by dashed lines), at the scar (May–June 1983), and after (after June 1983; A and C layers) El Niño, and a detail of primary and secondary lamellae within a cross-lamellar aragonite structure, showing differences in the organic matrix components during and after the event.

Shell samples were collected by commercial divers near Los Chimos (∼9.30^o^S), between Chimbote and Casma, in the coast of Peru [5] (Figure 1A), and purchased fresh in November of 1984. Three valves (2TP4-2, 2TP4-3 and 2TP4-4) of different specimens were used in this study, using the valve 2TP4-2 as reference based on a previous study [5] (Figure 1B). Based on growth rates previously estimated in these shells [2], [3], isotopic measurements [2], [5], and microscopic observations in this study (Figures 1B and S1), the boundaries between shell regions precipitated in relation to El Niño event can be determined with high precision. These shells reflect the time of maximum sea-surface temperature (SST) anomaly of El Niño in May-June of 1983, associated with a rise of nearly 10°C in SST [1], through the development of a scar [2], [3] (Figures 1 and S1). This scar represents a transition zone, of variable width between 5–15 mm, between the shell biomineralization conditions that occurred before and during El Niño, prior to the period May-June of 1983, and those after the event until capture in November of 1984 (Figure 1; Figures S1, S3 and S4).

Prior to elemental measurements by LA-ICP-MS and EPMA and chemico-structural observations by SEM and Raman spectroscopy, valves were rinsed in MilliQ (Millipore) water and air-dried. Subsequently, longitudinal sections of valves were embedded in epoxy resin for grinding and polishing. Then, the surfaces were ultra-polished, after grinding, initially with 6 and 3 micron thick diamond paste, with aluminium oxide (1 and 0.3 µm) and finally with colloidal silica (0.06 µm).

### Structural and Chemical Characterization

#### Scanning electron microscopy (SEM)

SEM observations were conducted after LA-ICP-MS analyses and Raman point measurements (Figures 1B and S1). This approach was taken to verify the location of individual LA-ICP-MS spot analyses (with an error of ±10 µm) in reference to different microstructural layers and shell regions precipitated during and after El Niño (example in Figure S1I). Samples were etched with 5% HCl acid for 30 seconds and gold coated. SEM and BSE observations were done using a JEOL 7000 field-emission scanning electron microscope (CAF – University of Alabama), under high vacuum and 20 kV.

#### Raman spectroscopy

**Figure 2:**
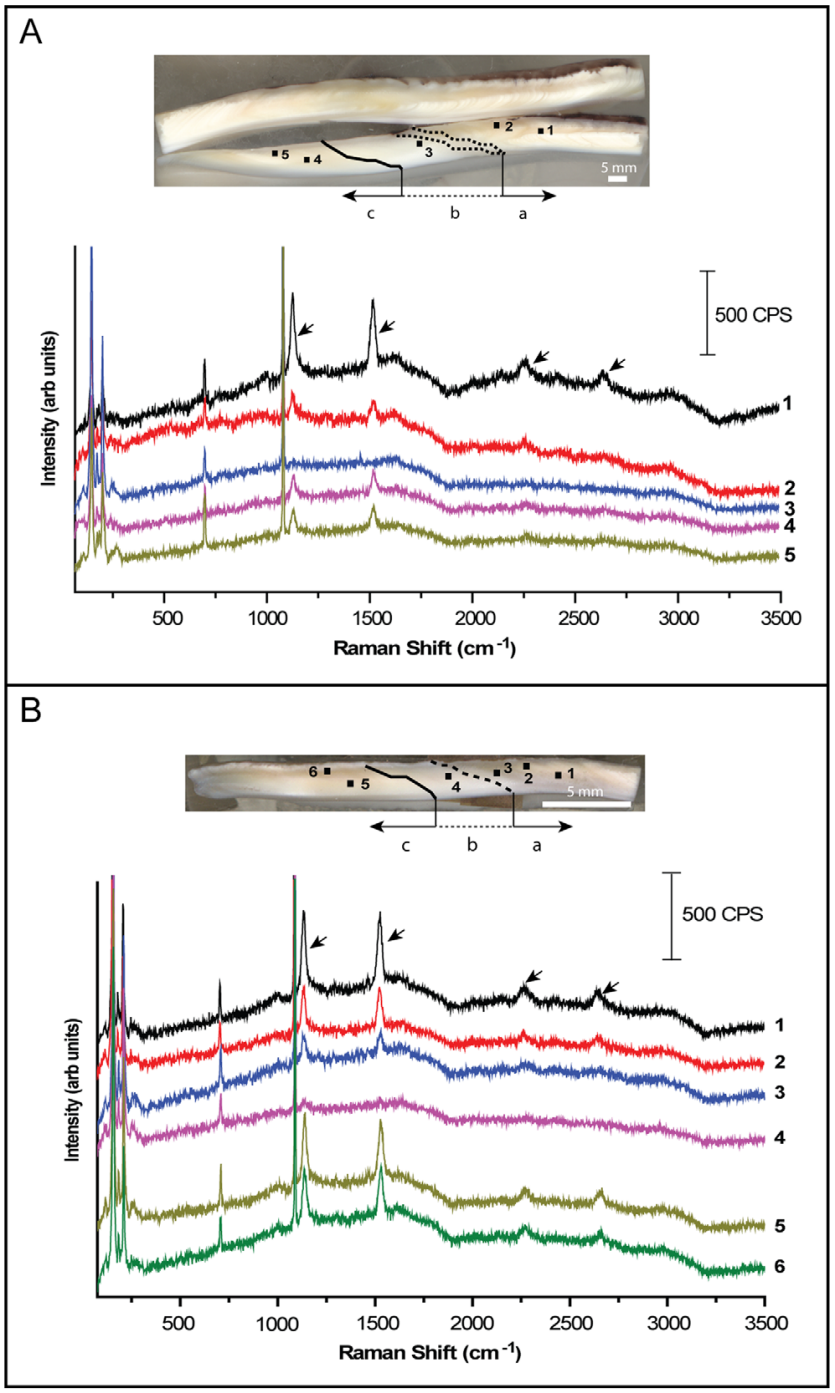
*In situ* Raman spectroscopy data. Specimens 2TP4-3 (A) and 2TP4-4 (B) showing shell regions precipitated during (a), around the scar (b), and after (c) the 1982–1983 El Niño event. Top image showing the shell with individual measurements with the shell scar (dashed lines) as a reference, and the bottom image with the corresponding Raman shift peaks. Peaks [arrows] at 1135 cm^−1^ and 1523 cm^−1^, due to (ν_2_) C-C single bonds and (ν_1_) C = C double bonds respectively, and the corresponding overtone bands at 2270 cm^−1^ and 2645 cm^−1^, disappear at the shell regions coincident with the maximum SST anomaly until normal biomineralization conditions resume after El Niño event.

*In situ* Raman analyses were performed with a Jobin-Yvon HR800 UV Raman Spectrometer, fitted with an argon-ion laser providing an excitation wavelength of 514 nm, in the Dept. of Chemistry of the University of Alabama. The interpretation of Raman peak data (Figure 2) is as follows: the two low wavenumber features, 156 cm^−1^ and 209 cm^−1^, are attributed to CaCO_3_ lattice vibrations, with the latter being specific to aragonite [12]. The peaks shown at 707 cm^−1^ and 1089 cm^−1^ are assigned as CaCO_3_ in-plane and symmetric vibrational modes respectively [13]. The peak present at 1135 cm^−1^ is due to (ν_2_) C-C single bonds and 1523 cm^−1^ to (ν_1_) C = C double bonds. The peak located at 2270 cm^−1^ is an (ν_2_) overtone band while the 2645 cm^−1^ peak is identified as a combination band of both (ν_2_) and (ν_1_) [14], [15]. These organic peaks (1135 cm^−1^, 1523 cm^−1^, 2270 cm^−1^, and 2645 cm^−1^) are related to polyene molecules that are associated in carbonate biomineral structures to pigment-protein complexes (chromo-proteins) [15]–[17].

#### Electron microprobe analysis (EPMA) mapping

Prior to LA-ICP-MS measurements, the distribution of several elements (Sr, Ca, Mg, Mn, S, Ba, and P) was mapped in three shells using a Cameca SX 100 instrument (15 kV; 1 nA; 180 microseconds per point) housed at the Natural History Museum in London. Besides Ca, only Mg and Sr were well above detection limits allowing the use of EPMA maps to characterize different shell regions in relation to the location of the scar (example in Figure S2).

#### Laser-ablation inductively-coupled mass spectrometry (LA-ICP-MS)

Elemental concentrations were calculated using the National Institute of Standards and Technology (NIST) standard reference material 612 for calibration and calcium (average 37.2 wt% calculated with ICP-OES and EPMA) was used for internal standardization. Also, in addition to the internal calibration, concentrations of Mg^2+^, Sr^2+^, and Ba^2+^ were calculated by ICP-OES [18] prior to LA-ICP-MS analyses to check accuracy, and it is better than 50 ppm, for all elements, in a comparison of ICP-OES and LA-ICP-MS data. The LA-ICP-MS technique employed an ESI (New Wave Research) UP193FX [193 nm wavelength, 2.5 J cm^−2^ fluence and 15 µm crater diameter] laser ablation accessory coupled to an Agilent 7500cs ICP-MS, with an acquisition duration of 90 s (ca 40 s background/50 s signal), at the Natural History Museum in London. For this study, signal intensities are shown for ^25^Mg, ^86^Sr, and ^137^Ba, and the relative standard deviation measurements are based on four measurements of the NIST 612 standard in each run [mean relative standard deviation -RSD (%) = 1.44 for analyzed elements, and with an overall RSD<2% for all analyses], and individual spot analyses of 20 µm (crater size). Average detection limits for each element are: 1.3 ppm (*n = 5*) for ^25^Mg, 4.1 ppm for ^86^Sr (*n = 5*), and 0.08 ppm (*n = 5*) for ^137^Ba. Data for elements (ppm) and Sr/Ca, Mg/Ca, and Ba/Ca (mmol/mol) are reported in Tables S1, S2, and S3.

## Results

Prior to the formation of the scar, the shell is characterized by the presence of three layers of cross-lamellar aragonite, with the middle layer showing growth lines, and primary and secondary lamellae enriched in intercrystalline organic matrix components (Figures 1B and S1). At the location of the scar, coincident with El Niño time of maximum SST anomaly, the middle layer disappears, by the thickening of lamellar structures, and aragonite crystallites are more clearly recognized because of the loss of organic coatings within the lamellae (Figure 1B). These microstructural changes are observed in all analyzed specimens. *In situ* high-resolution Raman spectroscopy measurements confirm the reduction of organic matrix components at the location of the shell scar (Figure 2). Peaks at 1135 cm^−1^ and 1523 cm^−1^, due to (ν_2_) C-C single bonds and (ν_1_) C = C double bonds respectively, and the corresponding overtone bands at 2270 cm^−1^ and 2645 cm^−1^, disappear at this transition zone until normal biomineralization conditions resume after the El Niño event (Figure 2).

**Figure 3:**
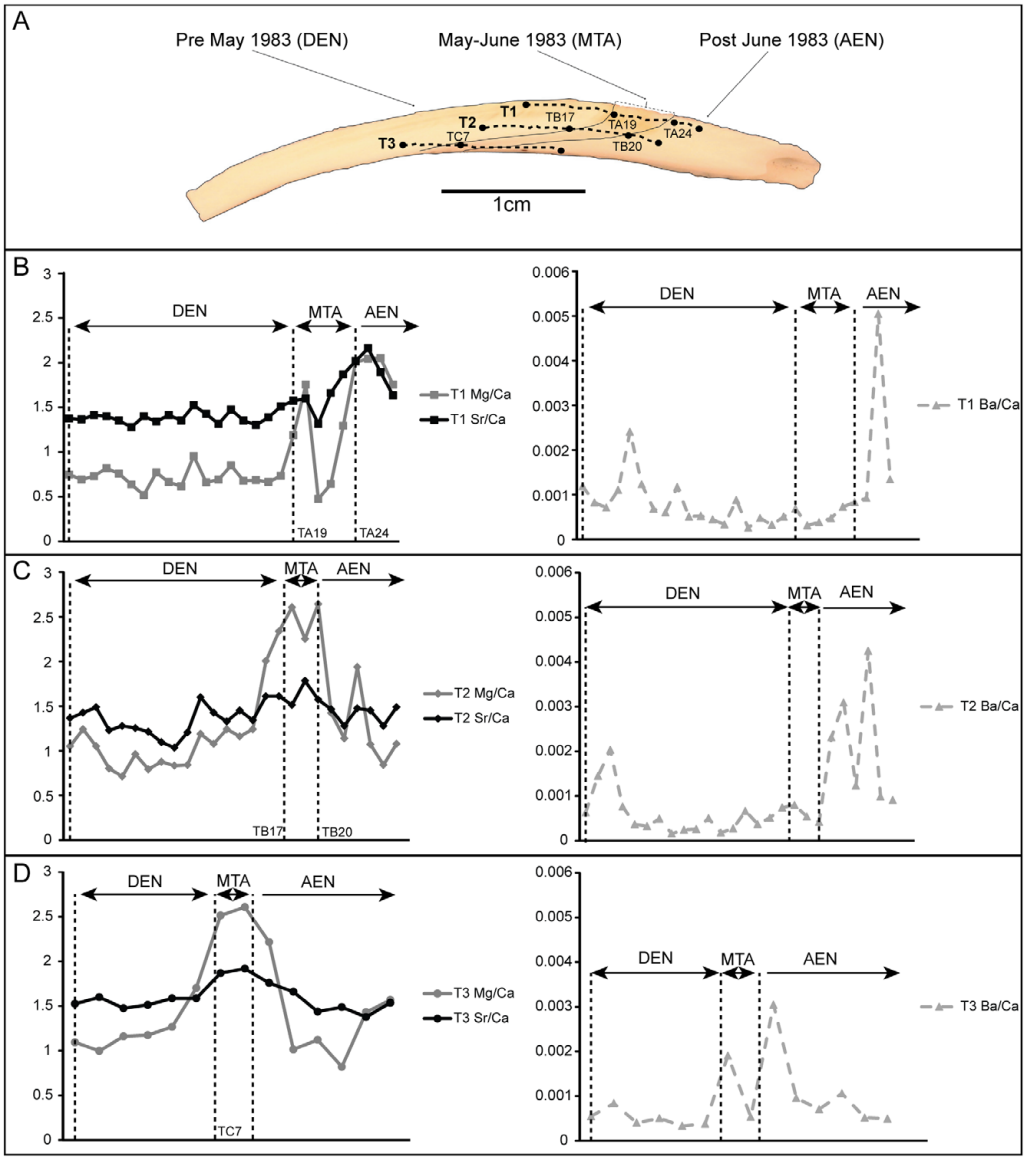
Profiles of Mg/Ca, Sr/Ca and Ba/Ca variability in shell transects of 2TP4-2 specimen during (DEN), at the transition zone – scar (MTA), and after (AEN) the 1982–1983 El Niño event. (A) Image of the shell in longitudinal section showing the location of transects, and some individual measurements as a reference, for each transect. (B) outer layer transect; (C) middle layer transect; (D) inner layer transect. [Sr/Ca - black solid line, Mg/Ca - grey solid line, and Ba/Ca – grey dashed line; number of individual measurements marked by solid squares (transect 1– outer layer), solid diamonds (transect 2– middle layer), and solid circles (transect 3– inner layer) for Mg/Ca and Sr/Ca, and solid triangles for Ba/Ca in all transects; mmol/mol units in all ‘y’ axes; error is <0.6% for all elemental ratios].

Variations in strontium, magnesium, and barium, previously assessed as environmental proxies in mollusk shells [9]–[11], were explored in relation to these biomineralization changes. Prior to *in situ* quantitative measurements, qualitative variations in trace elements were determined by electron probe microanalysis (EPMA) mapping (Figure S2). Strontium and magnesium were clearly above detection limits showing an increase near the shell scar indicative of the maximum SST anomaly. Although there is an enhancement in the signal of both elements, the strontium signal is only present at the scar while the magnesium concentration appears to increase prior to the scar as well (Figure S2). High-resolution Sr/Ca, Mg/Ca, and Ba/Ca profiles, by laser-ablation inductively-coupled mass spectrometry (LA-ICP-MS), were generated along the shell layers to test their potential utility as proxies for temperature and productivity changes associated with El Niño (Figure 3; Figures S3 and S4). The first observation is that there is a great variability of Me/Ca ratios, ranging, for example, from 1 to 2.7 mmol/mol in Mg/Ca and 1.3 to 2.3 mmol/mol in Sr/Ca, in different shell layers of the same specimen, and also in a comparison among different specimens (Figure 3; Figures S3 and S4). There are, however, common trends in the behavior of analyzed trace elements in shell regions corresponding to growth during El Niño (DEN), at the transition zone represented by the scar (maximum SST anomaly – MTA), and after El Niño (AEN; post June 1983) [1]. We observed quantifiable changes in Sr/Ca and Mg/Ca only at the location of the scar. Significant increase in Sr/Ca (p<0.01) is observed only in one transect across the outer layer of the specimen 2TP4-2 (Figure 3B); a Mann-Whitney similarity test [19], with a rejection level of 95%, indicates that Sr/Ca does not change significantly prior to and after the scar (p>0.5) within profiles of other shell layers of the same specimen (2TP4-2) and in a comparison among outer and middle shell layers of other specimens (Figure 3; Figures S3 and S4). In contrast, Mg/Ca values significantly increase (1–1.5 mmol/mol; p<0.01) at this transition zone in all shell layers of specimen 2TP4-2 (Figure 3), and in external and middle layers, used for comparison, of the other two specimens (2TP4-3 and 2TP-4; see Figures S3 and S4). Barium concentrations in these shells are much lower than strontium and magnesium (Tables S1, S2, and S3), but a quantifiable increase in Ba/Ca, between 0.02 and 0.05 mmol/mol, is present in shell regions of all specimens representing growth after El Niño (post-June 1983). In general, characteristic Ba/Ca peaks appear in profiles after the transition zone in contrast to Mg/Ca variations just at the location of the shell scar (Figure 3; Figures S3 and S4).

## Discussion

Our observations indicate that biomineralization changes in these shells are associated with a clear loss of intercrystalline organic components. Based on Raman data (Figure 2 and references), this loss is directly related to the modification of shell chromo-proteins at the moment of maximum SST anomaly linked to El Niño event, which is interpreted to be triggered by the protein denaturation connected to the significant increase of seawater temperature [1]. However, the change in shell chromo-proteins does not prevent these bivalves from producing biomineral structures. A possible explanation is linked to the fact that bivalve mollusks form their skeletons by precipitating crystals not from saturated solutions but from amorphous calcium carbonate (ACC) [20], [21]. Commonly, ACC precipitates into aragonite by water expulsion and the addition of proteins and double-charged ions (e.g., Mg^2+^ and Sr^2+^) [22]. In the absence of a fraction of the protein content, aragonite can be precipitated by incorporation of double-charged ions directly into ACC [20], [22]. Thus, these bivalves may be able to precipitate their aragonite shells by two different mechanisms during El Niño, in particular at the time of the maximum SST anomaly throughout the event. This explanation is also supported because of a higher proportion of sugars than glycoproteins in these cardioidean bivalves [23], helping to stabilize ACC, and it may explain the mortality of other bivalve species with a different sugar/glycoprotein proportion even within the same biomineral structures [23]. This finding confirms the long-established idea that the cooperation between proteins and polysaccharides is essential for carbonate biomineralization [24], and that the reduction, or loss of functionality, of only some proteins does not prevent carbonate biomineralization in some groups of bivalves.

Trace element variations in mollusk shells have been previously attributed to kinetic effects [25]–[26], calcification rates [26]–[28], and/or metabolic effects [27], [29]. Observed Sr^2+^ variations, with an increase in strontium content shown by probe mapping and Sr/Ca in a shell transect at the location of the scar, could be explained by lattice substitutions of Ca^2+^ by Sr^2+^, and related to the anomaly of nearly 10°C in SST associated with the 1982–83 El Niño. Such strontium variations are, however, difficult to quantify in these bivalve shells and not useful to detect such maximum seawater temperature variations linked to El Niño events. The main reason may be that constant Sr^2+^ concentrations are expected for aragonite [30]. Sr^2+^ is likely to be actively involved in aragonite precipitation as the main double-charged ion associated with proteins for ACC stabilization [20]. If a modification in shell mineral structure occurs with El Niño, Sr^+2^ incorporation into aragonite may be temporarily shifting from the lattice substitution to crystalline faces by sorption [31], but without a major change in strontium shell content. In contrast, predictable magnesium variations are recorded in all the analyzed shells. As a result, Mg/Ca can be used as a proxy to detect the timing of maximum SST anomaly of El Niño if its magnitude is sufficient to modify the shell organic matrix content, in particular the protein fraction. Although Mg/Ca has been argued to serve as a paleothermometer in calcite shells layers of some bivalves [10], the same explanation to link Mg/Ca and changes in seawater temperature may not apply here. A positive SST anomaly occurred throughout the event, approximately from September-October 1982 to June 1983 [1], but the Mg/Ca variation is only located in the shell region corresponding to the moment of maximum temperature change (May-June 1983). Additionally, recent studies have not found a correspondence between Mg/Ca and oxygen isotope data related to past temperature fluctuations on the recording of El Niño [8]. Furthermore, previous studies have found that magnesium does not seem to be a lattice component in aragonite bivalve shells [32]. However, observed biomineralization changes can explain the behavior of magnesium in these shells. Mg/Ca shell profiles prior to the location of the scar suggest relatively low quantities of Mg^2+^ that may have been incorporated into aragonite associated with proteins [20], [21]. At the scar, Mg/Ca increases, in parallel to the disappearance of intercrystalline organic matrix and chromo-proteins peaks in Raman spectra (Figure 2), indicating a possible intensification of Mg^2+^ incorporation into ACC for its stabilization [21], and in order to precipitate aragonite rapidly [33]. Rapid aragonite precipitation may be responsible for the observation of thickening in crossed-lamellar microstructures and the disappearance of growth lines in the shell (Figures 1 and S1). An inter-individual comparison of shells also demonstrates the presence of Ba/Ca peaks, mainly present in shell regions recording growth after El Niño (after June 1983) (Figure 3; Figures S3 and S4). These peaks represent an increase in barium content may be interpreted as a record in these shells of the recovery of primary productivity or a change in the content of dissolved barium in water on the Peruvian coast [9], after the decrease in productivity during El Niño [1]. These changes in barium may help then to determine the relative duration of these events as recorded in archaeological and paleontological bivalve shells if growth history is calculated from daily or subdaily growth increments.

### Conclusions

Results herein show that environmental perturbations associated with El Niño events can induce chemico-structural changes in mollusk shells, demonstrating a direct link between climatic conditions and biomineralization processes. The presence of a dual-mechanism of carbonate shell secretion during the event explains the survival of some bivalve taxa, while clarifying elemental proxy signals. Sr/Ca and Mg/Ca measurements do not seem to be useful as proxies to characterize SST anomalies related to El Niño in these surviving taxa. Although Sr/Ca temperature-proxy is successfully used in corals for the reconstruction of these events [34]–[36], any significant variation in Sr/Ca is difficult to detect in these bivalves. Meanwhile, an increase in Mg/Ca associated with the timing of the maximum SST anomaly is recorded, but related to the need of stabilization of ACC and rapid secretion of aragonite. Nevertheless, Mg/Ca does serve as a SST threshold proxy. In contrast, Ba/Ca is a potentialproxy for the recording of primary productivity or the amount of dissolved barium in water, and useful to detect the duration of El Niño, including the possibility of detecting the onset of these events when neither visible changes in shell structure nor other chemical changes consistently record that stage. Overall, our study contributes to a better understanding of marine invertebrate biomineralization under rapid environmental modifications associated with climate change, and the interpretation of elemental proxies recorded in bivalve shells. Further research may indicate that the dual-mechanism for carbonate shell secretion identified here arose as an evolutionary adaptation to temperature stress for some molluscan species [37]. As a consequence, a better understanding of evolution, latitudinal distribution, and past migration pathways of mollusk faunas along the Pacific coast of South America could be achieved in relation to El Niño events [37]–[39].

Finally, our results are also important in maximizing the utility of archaeological and paleontological bivalve specimens as paleoclimate archives. The Peruvian coast is a core region for observing the El Niño phase of ENSO (El Niño/Southern Oscillation), which plays a critical role in the interannual climatic variability. As a tropical desert coast fronted by a cool water current, this region lacks more usual sources of medium to high resolution paleoclimatic records; for example, lakes and corals are absent while deep ice from the high Andes responds to multiple climatic signals [40]. Biogeographic and isotopic data on mollusks from natural and cultural deposits, particularly over the ∼14,000 years people have inhabited Peru, provide the most detailed local records of near shore marine conditions [41]. While SST reconstructions from stable isotopes have provided conflicting values [42], the analysis of elemental proxies presented here will significantly aid in assessing the intensity and duration of paleo- El Niño events. Systematically employed, such analyses may help understand not only the past but also the future evolution of the ENSO system.

## Supporting Information

Figure S1 ***T. procerum*** **shells and detailed structure observations by scanning electron microscopy (SEM).** (**A**) Images of *T. procerum* valves with white arrows indicating the location of the scar associated to El Niño maximum SST anomaly [scale bar = 4 cm]. (**B**) Detail SEM image of the scar (white arrows) of the valve 2TP4-4 [scale bar = 10 µm]. (**C**) Cross-lamellar aragonite in the outer layer of the valve 2TP4-4 before the scar [scale bar = 10 µm]. (**D**) First and second order lamellae, without detail of characteristic cross-lamellar aragonite structure because of the high content in organic components, in the middle layer of the valve 2TP4-4 before the scar [scale bar = 10 µm]. (**E**) First and second order lamellae in the innermost layer of the valve 2TP4-4 before the scar [scale bar = 10 µm]. (**F**) Detail of (E) showing the lamellae coated with intercrystalline organic components [scale bar = 1 µm] (see also Figure 1 in the main text for comparison with data from the valve 2TP4-2). (**G**) First and second order lamellae in the innermost layer of the valve 2TP4-4 after the scar [scale bar = 10 µm]. (**H**) Detail of (G) showing the lamellae, without the coating of intercrystalline organic components as in (F) [scale bar = 1 µm] (see also Figure 1 in the main text for comparison with data from the valve 2TP4-2). (**I**) Example of the distribution of LA-ICP-MS individual spot analyses (arrows) in reference to shell layers and microstructure at the interface between the outer (OL) and middle (ML) layers, precipitated before the scar, in specimen 2TP4-2 [scale bar = 400 µm].
(TIF)

Figure S2 **Example of EPMA elemental maps along a longitudinal section of the valve 2TP4-4, with similar data obtained for valves 2TP4-2 and 2TP4-4 across the shell scar. (A, D)** BSE images showing the microstructure in the outer (A) and inner (D) shell regions and the location of the scar (dark line). (**B, E**) corresponding Sr maps to (A) and (D). (**C, F**) corresponding Mg maps to (A), (D), (B) and (E).
(TIF)

Figure S3 **Profiles of Mg/Ca, Sr/Ca and Ba/Ca variability in shell transects of 2TP4-3 specimen during (DEN), at the transition zone – scar (MTA), and after (AEN) the 1982–1983 El Niño event.** (**A**) Image of the shell longitudinal section showing the location of transects, and some individual measurements as a reference, for each transect. (**B**) Outer layer transect. (**C**) Middle layer transect. (**D**) Inner layer transect. [Sr/Ca - black solid line, Mg/Ca - grey solid line, and Ba/Ca – grey dashed line; number of individual measurements marked by solid squares (transect 1– outer layer), solid diamonds (transect 2– middle layer), and solid circles (transect 3– inner layer) for Mg/Ca and Sr/Ca, and solid triangles for Ba/Ca in all transects; mmol/mol units in all ‘y’ axes].
(TIF)

Figure S4 **Profiles of Mg/Ca, Sr/Ca and Ba/Ca variability in shell transects of 2TP4-4 specimen during (DEN), at the transition zone – scar (MTA), and after (AEN) the 1982–1983 El Niño event.** (**A**) Image of the shell longitudinal section showing the location of transects, and some individual measurements as a reference, for each transect. (**B**) Outer layer transect. (**C**) Middle layer transect. [Sr/Ca - black solid line, Mg/Ca - grey solid line, and Ba/Ca – grey dashed line; number of individual measurements marked by solid squares (transect 1– outer layer) and solid diamonds (transect 2– middle layer) for Mg/Ca and Sr/Ca, and solid triangles for Ba/Ca in all transects; mmol/mol units in all ‘y’ axes].
(TIF)

Table S1 LA-ICP-MS data for transects in shell 2TP4-2.
(DOCX)

Table S2 LA-ICP-MS data for transects in shell 2TP4-3.
(DOCX)

Table S3 LA-ICP-MS data for transects in shell 2TP4-4.
(DOCX)
