# Supplementary figures and images for: El Niño Impact on Mollusk Biomineralization–Implications for Trace Element Proxy Reconstructions and the Paleo-Archeological Record
Source: PLoS One. 2013 Feb 6;8(2):e54274. doi: 10.1371/journal.pone.0054274 (PMC3566134; doi:10.1371/journal.pone.0054274)

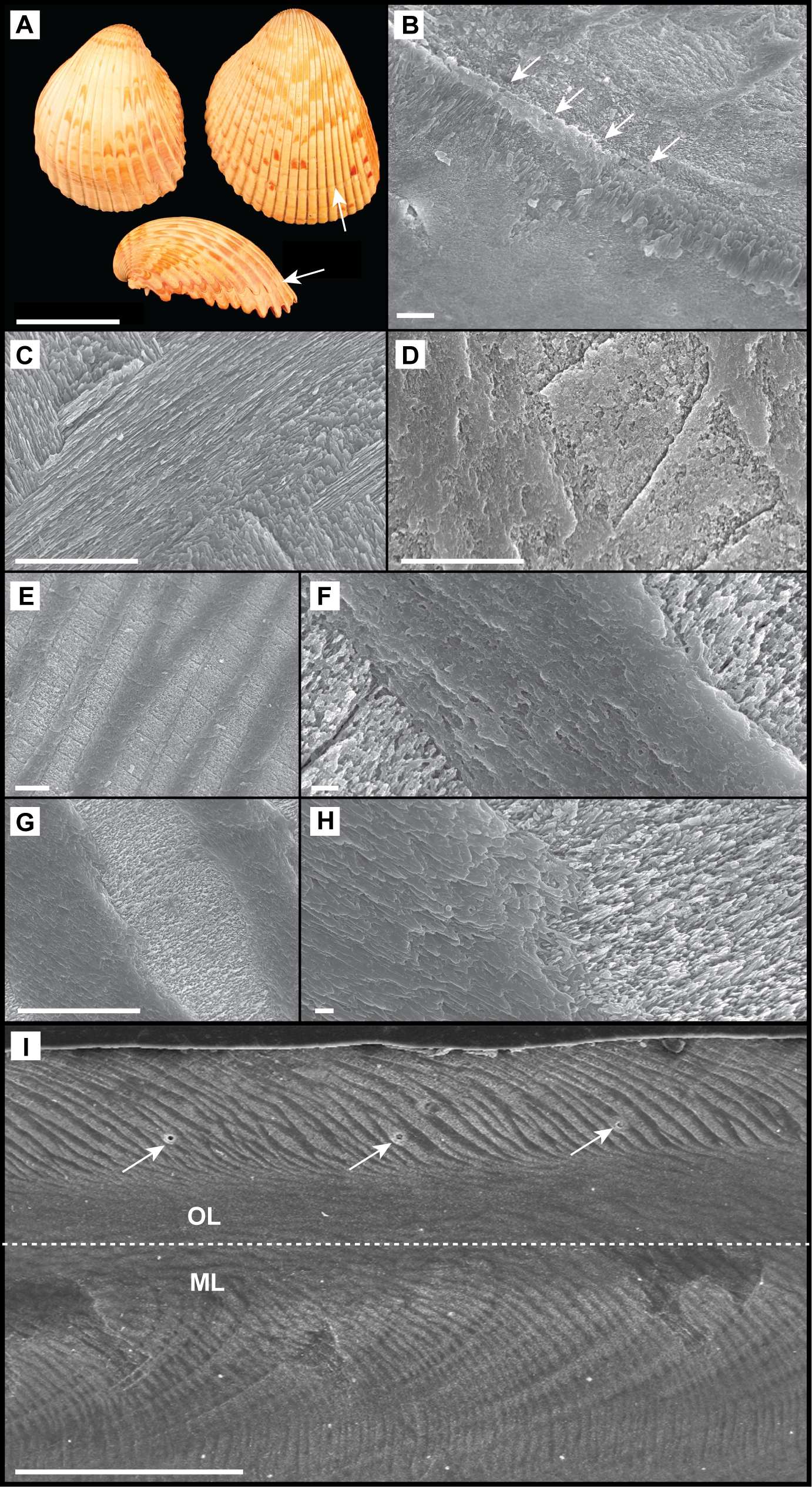

Supplement: Figure S1 — T. procerum shells and detailed structure observations by scanning electron microscopy (SEM). (A) Images of T. procerum valves with white arrows indicating the location of the scar associated to El Niño maximum SST anomaly [scale bar = 4 cm]. (B) Detail SEM image of the scar (white arrows) of the valve 2TP4-4 [scale bar = 10 µm]. (C) Cross-lamellar aragonite in the outer layer of the valve 2TP4-4 before the scar [scale bar = 10 µm]. (D) First and second order lamellae, without detail of characteristic cross-lamellar aragonite structure because of the high content in organic components, in the middle layer of the valve 2TP4-4 before the scar [scale bar = 10 µm]. (E) First and second order lamellae in the innermost layer of the valve 2TP4-4 before the scar [scale bar = 10 µm]. (F) Detail of (E) showing the lamellae coated with intercrystalline organic components [scale bar = 1 µm] (see also Figure 1 in the main text for comparison with data from the valve 2TP4-2). (G) First and second order lamellae in the innermost layer of the valve 2TP4-4 after the scar [scale bar = 10 µm]. (H) Detail of (G) showing the lamellae, without the coating of intercrystalline organic components as in (F) [scale bar = 1 µm] (see also Figure 1 in the main text for comparison with data from the valve 2TP4-2). (I) Example of the distribution of LA-ICP-MS individual spot analyses (arrows) in reference to shell layers and microstructure at the interface between the outer (OL) and middle (ML) layers, precipitated before the scar, in specimen 2TP4-2 [scale bar = 400 µm]. (TIF) [file pone.0054274.s001.tif]

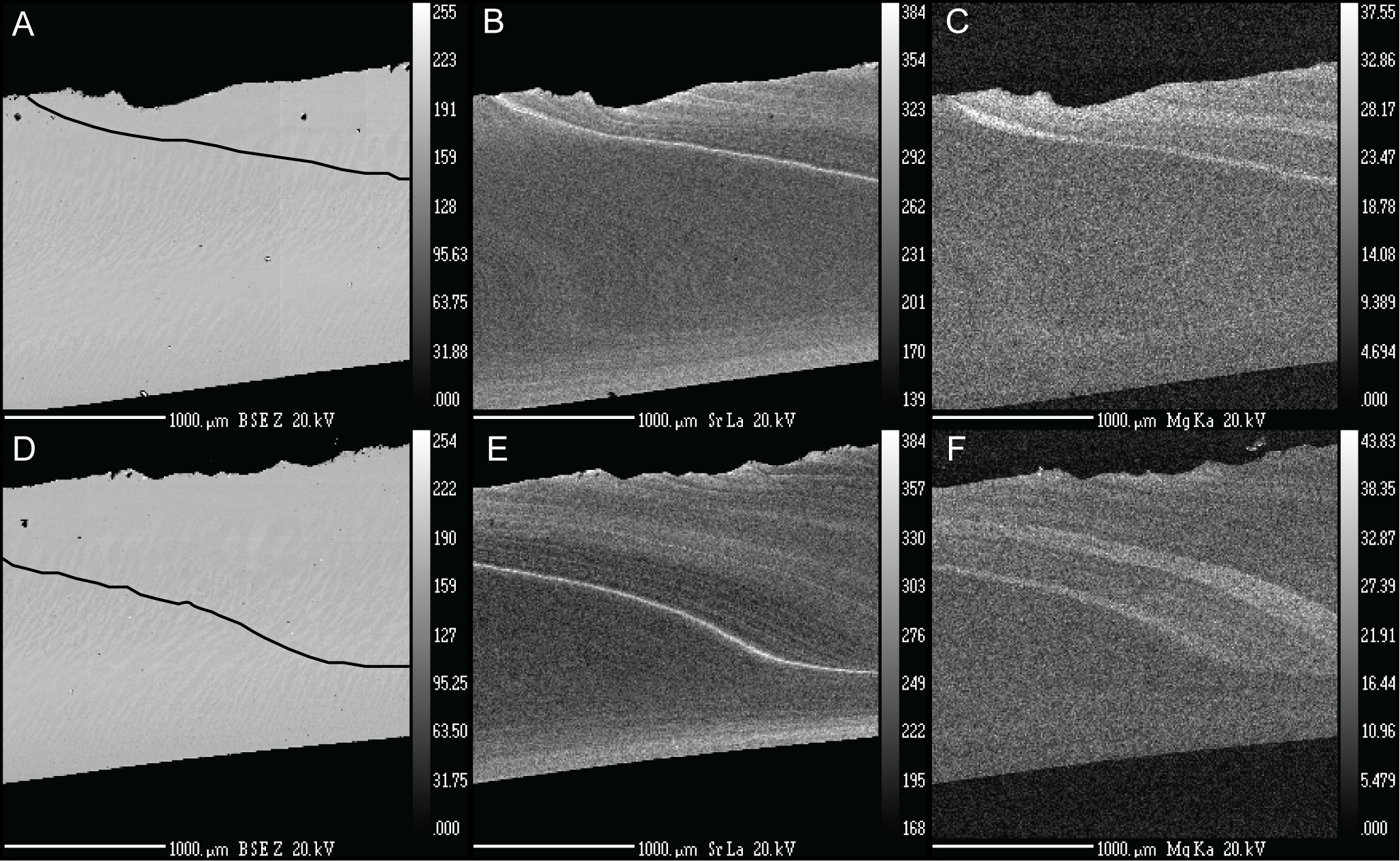

Supplement: Figure S2 — Example of EPMA elemental maps along a longitudinal section of the valve 2TP4-4, with similar data obtained for valves 2TP4-2 and 2TP4-4 across the shell scar. (A, D) BSE images showing the microstructure in the outer (A) and inner (D) shell regions and the location of the scar (dark line). (B, E) corresponding Sr maps to (A) and (D). (C, F) corresponding Mg maps to (A), (D), (B) and (E). (TIF) [file pone.0054274.s002.tif]

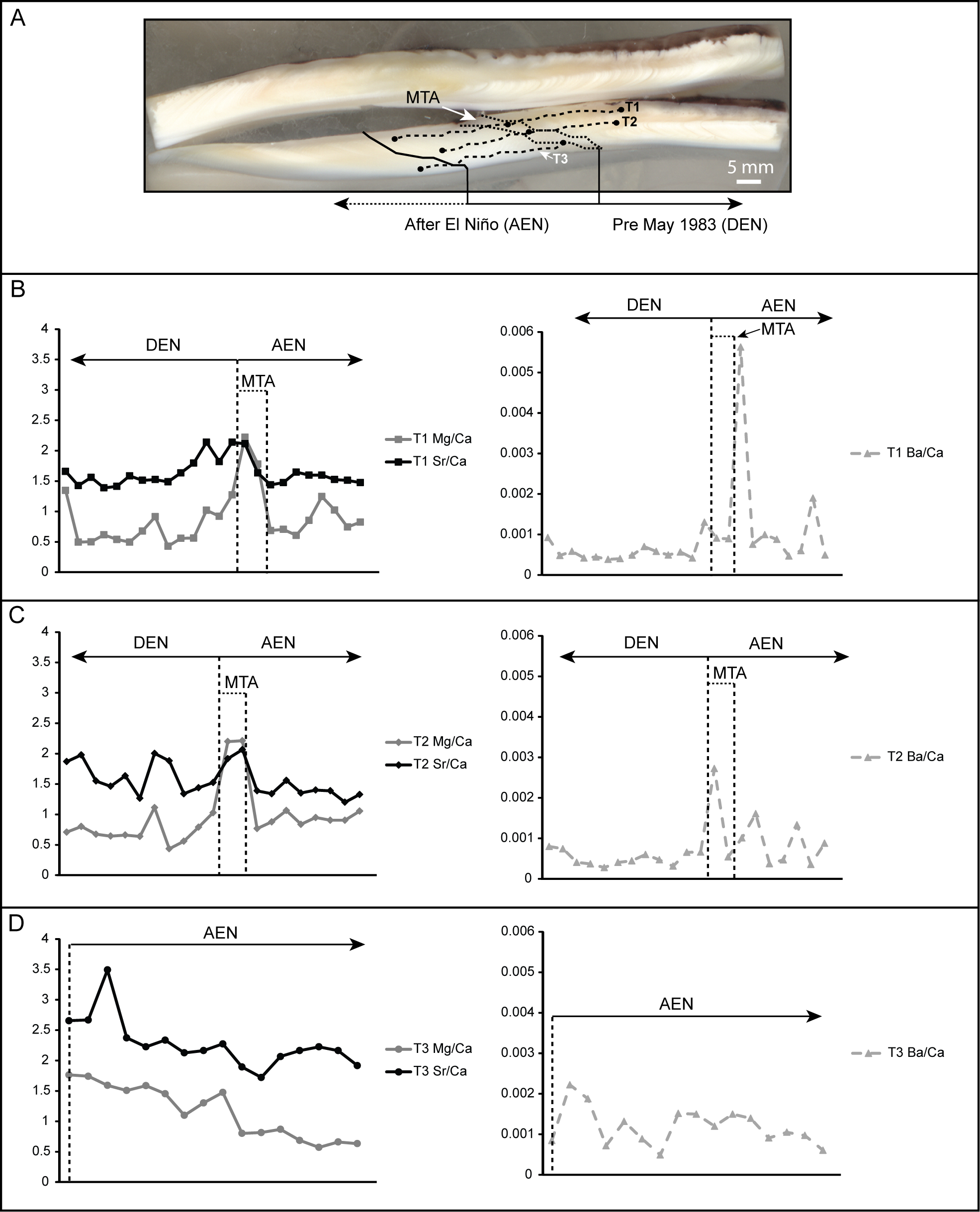

Supplement: Figure S3 — Profiles of Mg/Ca, Sr/Ca and Ba/Ca variability in shell transects of 2TP4-3 specimen during (DEN), at the transition zone – scar (MTA), and after (AEN) the 1982–1983 El Niño event. (A) Image of the shell longitudinal section showing the location of transects, and some individual measurements as a reference, for each transect. (B) Outer layer transect. (C) Middle layer transect. (D) Inner layer transect. [Sr/Ca - black solid line, Mg/Ca - grey solid line, and Ba/Ca – grey dashed line; number of individual measurements marked by solid squares (transect 1– outer layer), solid diamonds (transect 2– middle layer), and solid circles (transect 3– inner layer) for Mg/Ca and Sr/Ca, and solid triangles for Ba/Ca in all transects; mmol/mol units in all ‘y’ axes]. (TIF) [file pone.0054274.s003.tif]

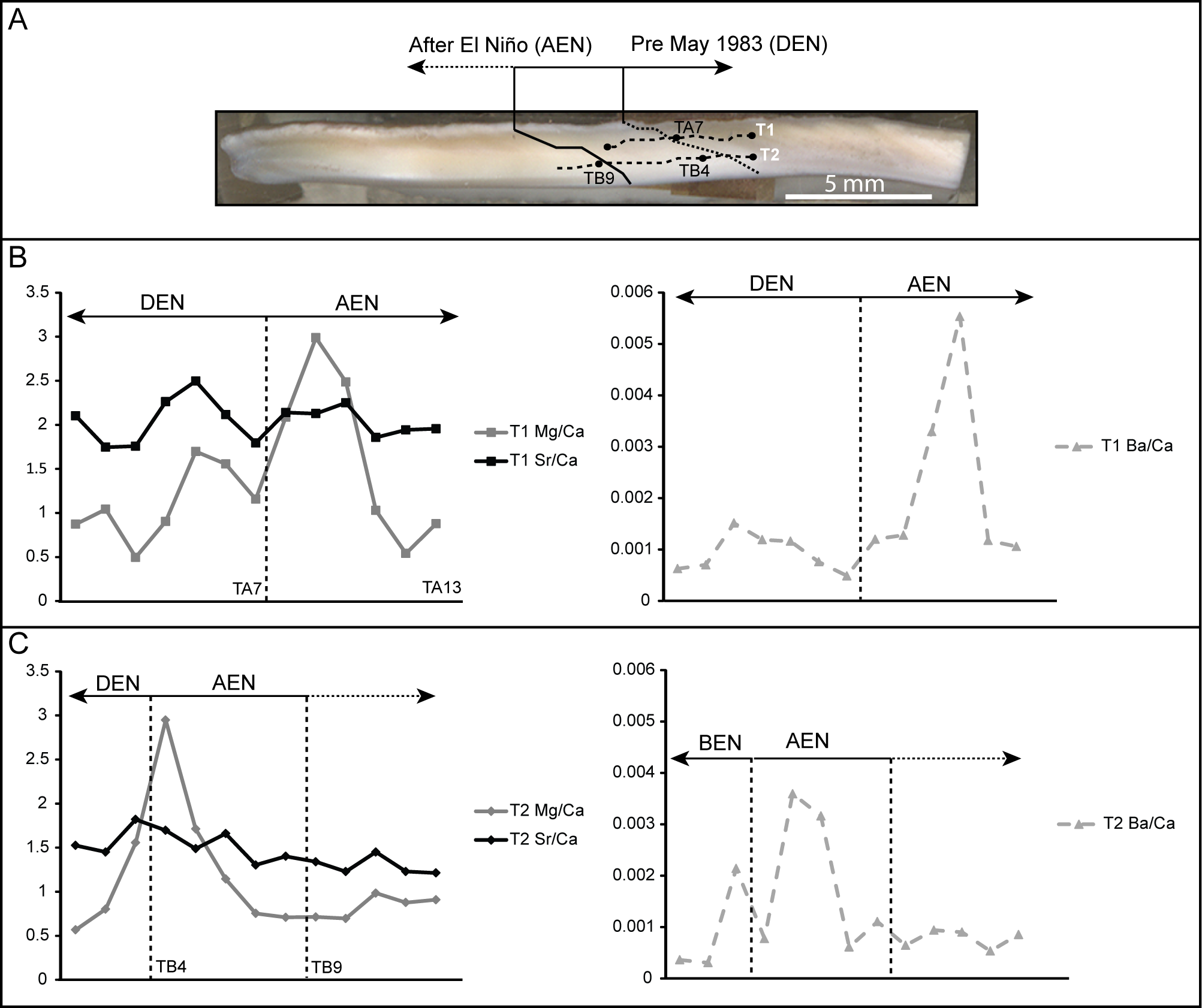

Supplement: Figure S4 — Profiles of Mg/Ca, Sr/Ca and Ba/Ca variability in shell transects of 2TP4-4 specimen during (DEN), at the transition zone – scar (MTA), and after (AEN) the 1982–1983 El Niño event. (A) Image of the shell longitudinal section showing the location of transects, and some individual measurements as a reference, for each transect. (B) Outer layer transect. (C) Middle layer transect. [Sr/Ca - black solid line, Mg/Ca - grey solid line, and Ba/Ca – grey dashed line; number of individual measurements marked by solid squares (transect 1– outer layer) and solid diamonds (transect 2– middle layer) for Mg/Ca and Sr/Ca, and solid triangles for Ba/Ca in all transects; mmol/mol units in all ‘y’ axes]. (TIF) [file pone.0054274.s004.tif]
