# Supplementary material for: El Niño Impact on Mollusk Biomineralization–Implications for Trace Element Proxy Reconstructions and the Paleo-Archeological Record
Source: PLoS One. 2013 Feb 6;8(2):e54274. doi: 10.1371/journal.pone.0054274 (PMC3566134; doi:10.1371/journal.pone.0054274)
Supplement: Table S1 — LA-ICP-MS data for transects in shell 2TP4-2. (DOCX) [file pone.0054274.s005.docx]

**Table S1**

|  | **Mg** | **Mg** | **Mg** | **CaO** | **Sr** | **Sr** | **Ba** | **Sample #** |
| --- | --- | --- | --- | --- | --- | --- | --- | --- |
| **Analysis ICPMS** | **24** | **25** | **26** | **43** | **86** | **88** | **137** |  |
|  | **ppm** | **ppm** | **ppm** | **wt%** | **ppm** | **ppm** | **ppm** |  |
| **mr24a03 03** | 168.00 | 169.00 | 168.00 | 53.20 | 1120.00 | 1110.00 | 1.52 | T1A1 |
| **mr24a04 04** | 167.00 | 156.00 | 166.00 | 53.20 | 1110.00 | 1080.00 | 1.05 | T1A2 |
| **mr24a05 05** | 163.00 | 165.00 | 168.00 | 53.20 | 1150.00 | 1100.00 | 0.91 | T1A3 |
| **mr24a06 06** | 178.00 | 185.00 | 177.00 | 53.20 | 1140.00 | 1130.00 | 1.41 | T1A4 |
| **mr24a07 07** | 167.00 | 171.00 | 162.00 | 53.20 | 1100.00 | 1130.00 | 3.07 | T1A5 |
| **mr24a08 08** | 152.00 | 144.00 | 136.00 | 53.20 | 1040.00 | 1060.00 | 1.57 | T1A6 |
| **mr24a09 09** | 131.00 | 117.00 | 130.00 | 53.20 | 1140.00 | 1130.00 | 0.87 | T1A7 |
| **mr24a10 10** | 165.00 | 174.00 | 158.00 | 53.20 | 1090.00 | 1090.00 | 0.77 | T1A8 |
| **mr24a11 11** | 151.00 | 150.00 | 165.00 | 53.20 | 1150.00 | 1140.00 | 1.49 | T1A9 |
| **mr24a12 12** | 142.00 | 139.00 | 142.00 | 53.20 | 1100.00 | 1060.00 | 0.65 | T1A10 |
| **mr24a13 13** | 223.00 | 216.00 | 233.00 | 53.20 | 1240.00 | 1220.00 | 0.67 | T1A11 |
| **mr24a14 14** | 156.00 | 149.00 | 162.00 | 53.20 | 1160.00 | 1100.00 | 0.57 | T1A12 |
| **mr24a15 15** | 163.00 | 156.00 | 149.00 | 53.20 | 1070.00 | 1050.00 | 0.42 | T1A13 |
| **mr24a16 16** | 206.00 | 192.00 | 217.00 | 53.20 | 1200.00 | 1180.00 | 1.12 | T1A14 |
| **mr24a17 17** | 173.00 | 153.00 | 170.00 | 53.20 | 1100.00 | 1070.00 | 0.33 | T1A15 |
| **mr24a18 18** | 161.00 | 155.00 | 154.00 | 53.20 | 1060.00 | 1080.00 | 0.61 | T1A16 |
| **mr24b03 03** | 146.00 | 150.00 | 143.00 | 53.20 | 1130.00 | 1110.00 | 0.42 | T1A17 |
| **mr24b04 04** | 166.00 | 166.00 | 154.00 | 53.20 | 1230.00 | 1230.00 | 0.65 | T1A18 |
| **mr24b05 05** | 261.00 | 268.00 | 245.00 | 53.20 | 1280.00 | 1290.00 | 0.88 | T1A19 |
| **mr24b06 06** | 389.00 | 396.00 | 373.00 | 53.20 | 1300.00 | 1290.00 | 0.40 | T1A20 |
| **mr24b07 07** | 116.00 | 107.00 | 99.20 | 53.20 | 1070.00 | 1100.00 | 0.49 | T1A21 |
| **mr24b08 08** | 148.00 | 145.00 | 154.00 | 53.20 | 1350.00 | 1370.00 | 0.60 | T1A22 |
| **mr24b09 09** | 274.00 | 292.00 | 271.00 | 53.20 | 1520.00 | 1520.00 | 0.93 | T1A23 |
| **mr24b10 10** | 462.00 | 453.00 | 448.00 | 53.20 | 1640.00 | 1650.00 | 1.06 | T1A24 |
| **mr24b11 11** | 474.00 | 461.00 | 483.00 | 53.20 | 1760.00 | 1800.00 | 1.18 | T1A25 |
| **mr24b12 12** | 458.00 | 463.00 | 465.00 | 53.20 | 1540.00 | 1510.00 | 6.43 | T1A26 |
| **mr24b13 13** | 391.00 | 396.00 | 409.00 | 53.20 | 1330.00 | 1290.00 | 1.71 | T1A27 |
| **mr24c03 03** | 230.00 | 237.00 | 238.00 | 53.20 | 1110.00 | 1090.00 | 0.81 | T2B1 |
| **mr24c04 04** | 275.00 | 280.00 | 279.00 | 53.20 | 1160.00 | 1140.00 | 1.85 | T2B2 |
| **mr24c05 05** | 247.00 | 237.00 | 227.00 | 53.20 | 1210.00 | 1260.00 | 2.59 | T2B3 |
| **mr24c06 06** | 184.00 | 181.00 | 201.00 | 53.20 | 1000.00 | 983.00 | 0.98 | T2B4 |
| **mr24c07 07** | 164.00 | 161.00 | 167.00 | 53.20 | 1040.00 | 987.00 | 0.47 | T2B5 |
| **mr24c08 08** | 215.00 | 217.00 | 223.00 | 53.20 | 1020.00 | 974.00 | 0.42 | T2B6 |
| **mr24c09 09** | 170.00 | 179.00 | 165.00 | 53.20 | 986.00 | 1000.00 | 0.63 | T2B7 |
| **mr24c10 10** | 210.00 | 198.00 | 211.00 | 53.20 | 891.00 | 846.00 | 0.20 | T2B8 |
| **mr24c11 11** | 197.00 | 188.00 | 210.00 | 53.20 | 842.00 | 874.00 | 0.31 | T2B9 |
| **mr24c12 12** | 203.00 | 190.00 | 202.00 | 53.20 | 981.00 | 964.00 | 0.33 | T2B10 |
| **mr24c13 13** | 279.00 | 268.00 | 277.00 | 53.20 | 1300.00 | 1220.00 | 0.64 | T2B11 |
| **mr24c14 14** | 254.00 | 243.00 | 252.00 | 53.20 | 1160.00 | 1110.00 | 0.23 | T2B12 |
| **mr24c15 15** | 267.00 | 280.00 | 276.00 | 53.20 | 1080.00 | 1030.00 | 0.35 | T2B13 |
| **mr24c16 16** | 272.00 | 262.00 | 274.00 | 53.20 | 1180.00 | 1120.00 | 0.85 | T2B14 |
| **mr24c17 17** | 296.00 | 280.00 | 292.00 | 53.20 | 1090.00 | 1070.00 | 0.48 | T2B15 |
| **mr24c18 18** | 484.00 | 452.00 | 484.00 | 53.20 | 1310.00 | 1290.00 | 0.66 | T2B16 |
| **mr24d03 03** | 559.00 | 527.00 | 551.00 | 53.20 | 1310.00 | 1320.00 | 0.95 | T2B17 |
| **mr24d04 04** | 590.00 | 588.00 | 589.00 | 53.20 | 1230.00 | 1220.00 | 1.02 | T2B18 |
| **mr24d05 05** | 500.00 | 508.00 | 481.00 | 53.20 | 1450.00 | 1380.00 | 0.69 | T2B19 |
| **mr24d06 06** | 562.00 | 596.00 | 548.00 | 53.20 | 1280.00 | 1240.00 | 0.53 | T2B20 |
| **mr24d07 07** | 315.00 | 321.00 | 324.00 | 53.20 | 1190.00 | 1130.00 | 2.95 | T2B21 |
| **mr24d08 08** | 253.00 | 257.00 | 263.00 | 53.20 | 1040.00 | 1040.00 | 3.94 | T2B22 |
| **mr24d09 09** | 422.00 | 437.00 | 420.00 | 53.20 | 1200.00 | 1150.00 | 1.57 | T2B23 |
| **mr24d10 10** | 249.00 | 242.00 | 236.00 | 53.20 | 1180.00 | 1160.00 | 5.42 | T2B24 |
| **mr24d11 11** | 205.00 | 190.00 | 206.00 | 53.20 | 1040.00 | 1040.00 | 1.26 | T2B25 |
| **mr24d12 12** | 249.00 | 243.00 | 249.00 | 53.20 | 1210.00 | 1170.00 | 1.16 | T2B26 |
| **mr24e16 16** | 240.00 | 247.00 | 228.00 | 53.20 | 1240.00 | 1240.00 | 0.70 | T3C1 |
| **mr24e15 15** | 230.00 | 225.00 | 237.00 | 53.20 | 1300.00 | 1290.00 | 1.07 | T3C2 |
| **mr24e14 14** | 256.00 | 262.00 | 271.00 | 53.20 | 1200.00 | 1170.00 | 0.52 | T3C3 |
| **mr24e13 13** | 265.00 | 265.00 | 276.00 | 53.20 | 1230.00 | 1160.00 | 0.64 | T3C4 |
| **mr24e03 03** | 289.00 | 286.00 | 281.00 | 53.20 | 1290.00 | 1300.00 | 0.42 | T3C5 |
| **mr24e04 04** | 393.00 | 384.00 | 384.00 | 53.20 | 1290.00 | 1270.00 | 0.48 | T3C6 |
| **mr24e05 05** | 553.00 | 567.00 | 562.00 | 53.20 | 1520.00 | 1510.00 | 2.43 | T3C7 |
| **mr24e06 06** | 629.00 | 588.00 | 611.00 | 53.20 | 1560.00 | 1490.00 | 0.68 | T3C8 |
| **mr24e07 07** | 483.00 | 500.00 | 481.00 | 53.20 | 1430.00 | 1410.00 | 3.88 | T3C9 |
| **mr24e08 08** | 241.00 | 229.00 | 238.00 | 53.20 | 1350.00 | 1310.00 | 1.22 | T3C10 |
| **mr24e09 09** | 256.00 | 253.00 | 247.00 | 53.20 | 1170.00 | 1130.00 | 0.90 | T3C11 |
| **mr24e10 10** | 202.00 | 185.00 | 196.00 | 53.20 | 1210.00 | 1180.00 | 1.35 | T3C12 |
| **mr24e11 11** | 328.00 | 323.00 | 328.00 | 53.20 | 1120.00 | 1090.00 | 0.66 | T3C13 |
| **mr24e12 12** | 339.00 | 354.00 | 336.00 | 53.20 | 1250.00 | 1220.00 | 0.63 | T3C14 |

**Table S1 (cont.).**

| **Sample #** | **Mg/Ca** | **Sr/Ca** | **B/Ca** |
| --- | --- | --- | --- |
|  | **(mmol/mol)** | **(mmol/mol)** | **(mmol/mol)** |
| T1A1 | 0.7491248 | 1.3771393 | 0.0011925 |
| T1A2 | 0.6914998 | 1.3648434 | 0.0008237 |
| T1A3 | 0.731394 | 1.414027 | 0.0007108 |
| T1A4 | 0.8200479 | 1.4017311 | 0.0011062 |
| T1A5 | 0.7579902 | 1.3525475 | 0.0024084 |
| T1A6 | 0.6383075 | 1.2787722 | 0.0012317 |
| T1A7 | 0.5186249 | 1.4017311 | 0.0006786 |
| T1A8 | 0.7712883 | 1.3402516 | 0.0006033 |
| T1A9 | 0.6649037 | 1.414027 | 0.0011689 |
| T1A10 | 0.6161441 | 1.3525475 | 0.0005099 |
| T1A11 | 0.9574613 | 1.5246899 | 0.0005256 |
| T1A12 | 0.660471 | 1.4263229 | 0.0004456 |
| T1A13 | 0.6914998 | 1.3156599 | 0.0003326 |
| T1A14 | 0.8510767 | 1.4755064 | 0.0008786 |
| T1A15 | 0.6782018 | 1.3525475 | 0.0002557 |
| T1A16 | 0.6870671 | 1.303364 | 0.000477 |
| T1A17 | 0.6649037 | 1.3894352 | 0.0003264 |
| T1A18 | 0.7358267 | 1.5123941 | 0.0005084 |
| T1A19 | 1.1879612 | 1.5738735 | 0.0006888 |
| T1A20 | 1.7553457 | 1.5984653 | 0.0003122 |
| T1A21 | 0.474298 | 1.3156599 | 0.0003813 |
| T1A22 | 0.6427402 | 1.6599447 | 0.0004723 |
| T1A23 | 1.2943458 | 1.8689748 | 0.0007265 |
| T1A24 | 2.0080091 | 2.0165254 | 0.0008316 |
| T1A25 | 2.0434706 | 2.1640761 | 0.0009257 |
| T1A26 | 2.052336 | 1.8935665 | 0.0050444 |
| T1A27 | 1.7553457 | 1.6353529 | 0.0013415 |
| T2B1 | 1.0505478 | 1.3648434 | 0.0006315 |
| T2B2 | 1.2411535 | 1.4263229 | 0.0014513 |
| T2B3 | 1.0505478 | 1.4878023 | 0.0020319 |
| T2B4 | 0.8023171 | 1.2295887 | 0.0007657 |
| T2B5 | 0.7136633 | 1.2787722 | 0.0003687 |
| T2B6 | 0.961894 | 1.2541804 | 0.0003256 |
| T2B7 | 0.7934517 | 1.2123744 | 0.0004927 |
| T2B8 | 0.8776729 | 1.0955635 | 0.0001593 |
| T2B9 | 0.8333459 | 1.0353137 | 0.0002463 |
| T2B10 | 0.8422113 | 1.2062265 | 0.0002597 |
| T2B11 | 1.1879612 | 1.5984653 | 0.0005037 |
| T2B12 | 1.077144 | 1.4263229 | 0.000182 |
| T2B13 | 1.2411535 | 1.3279558 | 0.0002738 |
| T2B14 | 1.1613651 | 1.4509146 | 0.0006653 |
| T2B15 | 1.2411535 | 1.3402516 | 0.0003726 |
| T2B16 | 2.0035764 | 1.6107612 | 0.0005139 |
| T2B17 | 2.3360283 | 1.6107612 | 0.0007421 |
| T2B18 | 2.6064224 | 1.5123941 | 0.0008002 |
| T2B19 | 2.2518071 | 1.7829036 | 0.0005429 |
| T2B20 | 2.6418839 | 1.5738735 | 0.0004189 |
| T2B21 | 1.4228939 | 1.4632105 | 0.0023143 |
| T2B22 | 1.1392016 | 1.2787722 | 0.003091 |
| T2B23 | 1.937086 | 1.4755064 | 0.0012317 |
| T2B24 | 1.0727113 | 1.4509146 | 0.004252 |
| T2B25 | 0.8422113 | 1.2787722 | 0.0009885 |
| T2B26 | 1.077144 | 1.4878023 | 0.00091 |
| T3C1 | 1.0948747 | 1.5246899 | 0.0005468 |
| T3C2 | 0.9973555 | 1.5984653 | 0.0008394 |
| T3C3 | 1.1613651 | 1.4755064 | 0.000404 |
| T3C4 | 1.1746632 | 1.5123941 | 0.0005044 |
| T3C5 | 1.2677497 | 1.5861694 | 0.0003326 |
| T3C6 | 1.7021534 | 1.5861694 | 0.0003797 |
| T3C7 | 2.5133359 | 1.8689748 | 0.0019064 |
| T3C8 | 2.6064224 | 1.9181583 | 0.0005343 |
| T3C9 | 2.2163456 | 1.7583118 | 0.0030439 |
| T3C10 | 1.0150863 | 1.6599447 | 0.0009571 |
| T3C11 | 1.1214709 | 1.4386187 | 0.0007068 |
| T3C12 | 0.8200479 | 1.4878023 | 0.0010591 |
| T3C13 | 1.4317593 | 1.3771393 | 0.000517 |
| T3C14 | 1.5691727 | 1.5369858 | 0.000495 |
