# Supplementary material for: El Niño Impact on Mollusk Biomineralization–Implications for Trace Element Proxy Reconstructions and the Paleo-Archeological Record
Source: PLoS One. 2013 Feb 6;8(2):e54274. doi: 10.1371/journal.pone.0054274 (PMC3566134; doi:10.1371/journal.pone.0054274)
Supplement: Table S2 — LA-ICP-MS data for transects in shell 2TP4-3. (DOCX) [file pone.0054274.s006.docx]

**Table S2**

|  | **Mg** | **Mg** | **Mg** | **CaO** | **Sr** | **Sr** | **Ba** | **Sample #** |
| --- | --- | --- | --- | --- | --- | --- | --- | --- |
| **Analysis ICPMS** | **24** | **25** | **26** | **43** | **86** | **88** | **137** |  |
|  | **ppm** | **ppm** | **ppm** | **wt%** | **ppm** | **ppm** | **ppm** |  |
| **mr21d03 03** | 286.00 | 304.00 | 266.00 | 53.20 | 1350.00 | 1330.00 | 1.18 | T1A1 |
| **mr21d04 04** | 120.00 | 112.00 | 120.00 | 53.20 | 1160.00 | 1130.00 | 0.62 | T1A2 |
| **mr21d05 05** | 108.00 | 113.00 | 103.00 | 53.20 | 1270.00 | 1250.00 | 0.75 | T1A3 |
| **mr21d06 06** | 140.00 | 139.00 | 143.00 | 53.20 | 1130.00 | 1130.00 | 0.54 | T1A4 |
| **mr21d07 07** | 121.00 | 122.00 | 114.00 | 53.20 | 1150.00 | 1130.00 | 0.57 | T1A5 |
| **mr21d08 08** | 121.00 | 112.00 | 134.00 | 53.20 | 1290.00 | 1310.00 | 0.49 | T1A6 |
| **mr21d09 09** | 149.00 | 153.00 | 147.00 | 53.20 | 1230.00 | 1240.00 | 0.52 | T1A7 |
| **mr21d10 10** | 209.00 | 206.00 | 216.00 | 53.20 | 1240.00 | 1240.00 | 0.63 | T1A8 |
| **mr21d11 11** | 102.00 | 96.50 | 91.10 | 53.20 | 1210.00 | 1200.00 | 0.89 | T1A9 |
| **mr21d12 12** | 130.00 | 126.00 | 118.00 | 53.20 | 1330.00 | 1320.00 | 0.74 | T1A10 |
| **mr21d13 13** | 125.00 | 127.00 | 122.00 | 53.20 | 1460.00 | 1380.00 | 0.63 | T1A11 |
| **mr21d14 14** | 231.00 | 230.00 | 232.00 | 53.20 | 1740.00 | 1600.00 | 0.73 | T1A12 |
| **mr21d15 15** | 214.00 | 208.00 | 219.00 | 53.20 | 1480.00 | 1500.00 | 0.53 | T1A13 |
| **mr21d16 16** | 272.00 | 287.00 | 280.00 | 53.20 | 1740.00 | 1640.00 | 1.66 | T1A14 |
| **mr21d17 17** | 509.00 | 501.00 | 505.00 | 53.20 | 1720.00 | 1720.00 | 1.16 | T1A15 |
| **mr21d18 18** | 389.00 | 402.00 | 391.00 | 53.20 | 1330.00 | 1310.00 | 1.14 | T1A16 |
| **mr21e03 03** | 149.00 | 155.00 | 155.00 | 53.20 | 1170.00 | 1100.00 | 7.17 | T1A17 |
| **mr21e04 04** | 161.00 | 159.00 | 163.00 | 53.20 | 1200.00 | 1130.00 | 0.97 | T1A18 |
| **mr21e05 05** | 135.00 | 137.00 | 143.00 | 53.20 | 1340.00 | 1250.00 | 1.27 | T1A19 |
| **mr21e06 06** | 194.00 | 193.00 | 183.00 | 53.20 | 1300.00 | 1270.00 | 1.12 | T1A20 |
| **mr21e07 07** | 277.00 | 281.00 | 287.00 | 53.20 | 1300.00 | 1240.00 | 0.60 | T1A21 |
| **mr21e08 08** | 228.00 | 231.00 | 231.00 | 53.20 | 1240.00 | 1210.00 | 0.77 | T1A22 |
| **mr21e09 09** | 168.00 | 168.00 | 172.00 | 53.20 | 1230.00 | 1230.00 | 2.42 | T1A23 |
| **mr21e10 10** | 195.00 | 186.00 | 189.00 | 53.20 | 1200.00 | 1180.00 | 0.63 | T1A24 |
| **mr21e11 11** | 168.00 | 160.00 | 173.00 | 53.20 | 1520.00 | 1500.00 | 1.02 | T2B1 |
| **mr21e12 12** | 169.00 | 181.00 | 167.00 | 53.20 | 1610.00 | 1590.00 | 0.95 | T2B2 |
| **mr21e13 13** | 154.00 | 152.00 | 151.00 | 53.20 | 1260.00 | 1230.00 | 0.52 | T2B3 |
| **mr21e14 14** | 150.00 | 145.00 | 131.00 | 53.20 | 1190.00 | 1180.00 | 0.47 | T2B4 |
| **mr21e15 15** | 157.00 | 149.00 | 148.00 | 53.20 | 1330.00 | 1320.00 | 0.36 | T2B5 |
| **mr21e16 16** | 142.00 | 144.00 | 144.00 | 53.20 | 1030.00 | 1070.00 | 0.52 | T2B6 |
| **mr21e17 17** | 253.00 | 251.00 | 248.00 | 53.20 | 1630.00 | 1630.00 | 0.57 | T2B7 |
| **mr21e18 18** | 107.00 | 98.10 | 110.00 | 53.20 | 1530.00 | 1540.00 | 0.77 | T2B8 |
| **mr21f03 03** | 120.00 | 126.00 | 113.00 | 53.20 | 1090.00 | 1080.00 | 0.60 | T2B9 |
| **mr21f04 04** | 183.00 | 178.00 | 176.00 | 53.20 | 1170.00 | 1160.00 | 0.40 | T2B10 |
| **mr21f05 05** | 227.00 | 232.00 | 204.00 | 53.20 | 1240.00 | 1280.00 | 0.84 | T2B11 |
| **mr21f06 06** | 526.00 | 496.00 | 508.00 | 53.20 | 1560.00 | 1580.00 | 0.85 | T2B12 |
| **mr21f07 07** | 530.00 | 499.00 | 479.00 | 53.20 | 1680.00 | 1730.00 | 3.47 | T2B13 |
| **mr21f08 08** | 190.00 | 173.00 | 169.00 | 53.20 | 1130.00 | 1130.00 | 0.69 | T2B14 |
| **mr21f09 09** | 206.00 | 198.00 | 192.00 | 53.20 | 1090.00 | 1070.00 | 1.29 | T2B15 |
| **mr21f10 10** | 229.00 | 240.00 | 232.00 | 53.20 | 1270.00 | 1270.00 | 2.05 | T2B16 |
| **mr21f11 11** | 206.00 | 189.00 | 196.00 | 53.20 | 1100.00 | 1050.00 | 0.48 | T2B17 |
| **mr21f12 12** | 217.00 | 214.00 | 209.00 | 53.20 | 1140.00 | 1120.00 | 0.60 | T2B18 |
| **mr21f13 13** | 201.00 | 204.00 | 200.00 | 53.20 | 1130.00 | 1120.00 | 1.70 | T2B19 |
| **mr21f14 14** | 227.00 | 204.00 | 200.00 | 53.20 | 976.00 | 1000.00 | 0.45 | T2B20 |
| **mr21f15 15** | 231.00 | 238.00 | 217.00 | 53.20 | 1080.00 | 998.00 | 1.12 | T2B21 |
| **mr22a03 03** | 385.00 | 398.00 | 383.00 | 53.20 | 2160.00 | 2100.00 | 1.08 | T3C1 |
| **mr22a04 04** | 409.00 | 393.00 | 399.00 | 53.20 | 2170.00 | 2220.00 | 2.83 | T3C2 |
| **mr22a05 05** | 344.00 | 359.00 | 367.00 | 53.20 | 2840.00 | 2680.00 | 2.39 | T3C3 |
| **mr22a06 06** | 333.00 | 340.00 | 343.00 | 53.20 | 1930.00 | 1940.00 | 0.92 | T3C4 |
| **mr22a07 07** | 359.00 | 358.00 | 318.00 | 53.20 | 1810.00 | 1730.00 | 1.68 | T3C5 |
| **mr22a08 08** | 308.00 | 328.00 | 305.00 | 53.20 | 1900.00 | 1820.00 | 1.13 | T3C6 |
| **mr22a09 09** | 266.00 | 248.00 | 282.00 | 53.20 | 1730.00 | 1680.00 | 0.63 | T3C7 |
| **mr22a10 10** | 296.00 | 294.00 | 290.00 | 53.20 | 1760.00 | 1840.00 | 1.93 | T3C8 |
| **mr22a11 11** | 314.00 | 333.00 | 294.00 | 53.20 | 1850.00 | 1860.00 | 1.91 | T3C9 |
| **mr22a12 12** | 182.00 | 181.00 | 181.00 | 53.20 | 1540.00 | 1450.00 | 1.53 | T3C10 |
| **mr22a13 13** | 182.00 | 184.00 | 191.00 | 53.20 | 1400.00 | 1390.00 | 1.91 | T3C11 |
| **mr22a14 14** | 198.00 | 196.00 | 196.00 | 53.20 | 1680.00 | 1680.00 | 1.78 | T3C12 |
| **mr22a15 15** | 147.00 | 155.00 | 168.00 | 53.20 | 1760.00 | 1790.00 | 1.16 | T3C13 |
| **mr22a16 16** | 148.00 | 129.00 | 155.00 | 53.20 | 1810.00 | 1820.00 | 1.34 | T3C14 |
| **mr22a17 17** | 152.00 | 149.00 | 164.00 | 53.20 | 1760.00 | 1740.00 | 1.24 | T3C15 |
| **mr22a18 18** | 145.00 | 143.00 | 141.00 | 53.20 | 1560.00 | 1570.00 | 0.77 | T3C16 |

**Table S2 (cont.)**

| **Sample #** | **Mg/Ca** | **Sr/Ca** | **Ba/Ca** |
| --- | --- | --- | --- |
|  | **mmol/mol** | **mmol/mol** | **mmol/mol** |
| T1A1 | 1.347538119 | 1.659944703 | 0.00092572 |
| T1A2 | 0.496461412 | 1.426322856 | 0.000484042 |
| T1A3 | 0.500894103 | 1.561577609 | 0.000586028 |
| T1A4 | 0.616144074 | 1.389435195 | 0.000421281 |
| T1A5 | 0.540788324 | 1.414026969 | 0.00044717 |
| T1A6 | 0.496461412 | 1.586169382 | 0.000387547 |
| T1A7 | 0.678201751 | 1.512394062 | 0.000404022 |
| T1A8 | 0.913134383 | 1.524689949 | 0.000490318 |
| T1A9 | 0.427754699 | 1.487802289 | 0.000701351 |
| T1A10 | 0.558519089 | 1.635352929 | 0.00058289 |
| T1A11 | 0.56295178 | 1.795199456 | 0.00049424 |
| T1A12 | 1.019518971 | 2.139484283 | 0.000569553 |
| T1A13 | 0.921999766 | 1.819791229 | 0.000418143 |
| T1A14 | 1.272182369 | 2.139484283 | 0.001302284 |
| T1A15 | 2.220778281 | 2.11489251 | 0.00091003 |
| T1A16 | 1.781941855 | 1.635352929 | 0.00089434 |
| T1A17 | 0.687067133 | 1.438618742 | 0.005624926 |
| T1A18 | 0.704797898 | 1.475506402 | 0.000759404 |
| T1A19 | 0.607278692 | 1.647648816 | 0.000996326 |
| T1A20 | 0.855509398 | 1.598465269 | 0.00087865 |
| T1A21 | 1.245586222 | 1.598465269 | 0.000469136 |
| T1A22 | 1.023951663 | 1.524689949 | 0.000603287 |
| T1A23 | 0.744692118 | 1.512394062 | 0.001898511 |
| T1A24 | 0.82448056 | 1.475506402 | 0.000492671 |
| T2B1 | 0.709230589 | 1.868974776 | 0.000800199 |
| T2B2 | 0.802317104 | 1.979637756 | 0.000744499 |
| T2B3 | 0.673769059 | 1.549281722 | 0.000410298 |
| T2B4 | 0.642740221 | 1.463210516 | 0.000371073 |
| T2B5 | 0.660470986 | 1.635352929 | 0.000279285 |
| T2B6 | 0.63830753 | 1.266476329 | 0.000407944 |
| T2B7 | 1.112605486 | 2.00422953 | 0.00044717 |
| T2B8 | 0.434847005 | 1.881270663 | 0.000602503 |
| T2B9 | 0.558519089 | 1.340251649 | 0.000470705 |
| T2B10 | 0.78901903 | 1.438618742 | 0.000314588 |
| T2B11 | 1.028384354 | 1.524689949 | 0.000655849 |
| T2B12 | 2.198614825 | 1.918158323 | 0.00066291 |
| T2B13 | 2.211912899 | 2.065708963 | 0.002722245 |
| T2B14 | 0.766855574 | 1.389435195 | 0.00054288 |
| T2B15 | 0.877672854 | 1.340251649 | 0.001012016 |
| T2B16 | 1.063845883 | 1.561577609 | 0.001608243 |
| T2B17 | 0.837778633 | 1.352547535 | 0.000376564 |
| T2B18 | 0.948595913 | 1.401731082 | 0.000469136 |
| T2B19 | 0.904269001 | 1.389435195 | 0.001333665 |
| T2B20 | 0.904269001 | 1.200078541 | 0.000355382 |
| T2B21 | 1.054980501 | 1.327955762 | 0.00087865 |
| T3C1 | 1.76421109 | 2.655911524 | 0.000847269 |
| T3C2 | 1.742047634 | 2.668207411 | 0.002220159 |
| T3C3 | 1.591336134 | 3.492031819 | 0.001874975 |
| T3C4 | 1.507115001 | 2.37310613 | 0.00071861 |
| T3C5 | 1.586903443 | 2.22555549 | 0.001317974 |
| T3C6 | 1.453922707 | 2.33621847 | 0.000886495 |
| T3C7 | 1.099307413 | 2.127188397 | 0.000490318 |
| T3C8 | 1.303211207 | 2.164076057 | 0.001514102 |
| T3C9 | 1.476086163 | 2.274739037 | 0.001498411 |
| T3C10 | 0.802317104 | 1.89356655 | 0.001200298 |
| T3C11 | 0.815615177 | 1.721424136 | 0.001498411 |
| T3C12 | 0.868807471 | 2.065708963 | 0.001396425 |
| T3C13 | 0.687067133 | 2.164076057 | 0.00091003 |
| T3C14 | 0.571817162 | 2.22555549 | 0.001051241 |
| T3C15 | 0.660470986 | 2.164076057 | 0.000972791 |
| T3C16 | 0.633874839 | 1.918158323 | 0.00060721 |
