# Supplementary material for: El Niño Impact on Mollusk Biomineralization–Implications for Trace Element Proxy Reconstructions and the Paleo-Archeological Record
Source: PLoS One. 2013 Feb 6;8(2):e54274. doi: 10.1371/journal.pone.0054274 (PMC3566134; doi:10.1371/journal.pone.0054274)
Supplement: Table S3 — LA-ICP-MS data for transects in shell 2TP4-4. (DOCX) [file pone.0054274.s007.docx]

**Table S3**

|  | **Mg** | **Mg** | **Mg** | **CaO** | **Sr** | **Sr** | **Ba** | **Sample #** |
| --- | --- | --- | --- | --- | --- | --- | --- | --- |
| **Analysis ICPMS** | **24** | **25** | **26** | **43** | **86** | **88** | **137** |  |
|  | **ppm** | **ppm** | **ppm** | **wt%** | **ppm** | **ppm** | **ppm** |  |
| **mr22b03 03** | 198.00 | 197.00 | 194.00 | 53.20 | 1710.00 | 1600.00 | 0.80 | T1A1 |
| **mr22b04 04** | 236.00 | 235.00 | 218.00 | 53.20 | 1420.00 | 1410.00 | 0.90 | T1A2 |
| **mr22b05 05** | 104.00 | 112.00 | 106.00 | 53.20 | 1430.00 | 1470.00 | 1.93 | T1A3 |
| **mr22b06 06** | 218.00 | 204.00 | 216.00 | 53.20 | 1840.00 | 1820.00 | 1.52 | T1A4 |
| **mr22b07 07** | 380.00 | 383.00 | 356.00 | 53.20 | 2030.00 | 1970.00 | 1.48 | T1A5 |
| **mr22b08 08** | 336.00 | 351.00 | 332.00 | 53.20 | 1720.00 | 1650.00 | 0.97 | T1A6 |
| **mr22b09 09** | 269.00 | 261.00 | 281.00 | 53.20 | 1460.00 | 1470.00 | 0.62 | T1A7 |
| **mr22b10 10** | 454.00 | 471.00 | 455.00 | 53.20 | 1740.00 | 1720.00 | 1.53 | T1A8 |
| **mr22b11 11** | 643.00 | 674.00 | 617.00 | 53.20 | 1730.00 | 1780.00 | 1.63 | T1A9 |
| **mr22b12 12** | 531.00 | 561.00 | 520.00 | 53.20 | 1830.00 | 1800.00 | 4.20 | T1A10 |
| **mr22b13 13** | 226.00 | 232.00 | 220.00 | 53.20 | 1510.00 | 1500.00 | 7.05 | T1A11 |
| **mr22b14 14** | 124.00 | 122.00 | 110.00 | 53.20 | 1580.00 | 1490.00 | 1.50 | T1A12 |
| **mr22b15 15** | 178.00 | 198.00 | 176.00 | 53.20 | 1590.00 | 1560.00 | 1.35 | T1A13 |
| **mr22c03 03** | 128.00 | 128.00 | 125.00 | 53.20 | 1240.00 | 1180.00 | 0.46 | T1B1 |
| **mr22c04 04** | 181.00 | 181.00 | 188.00 | 53.20 | 1180.00 | 1190.00 | 0.39 | T1B2 |
| **mr22c05 05** | 352.00 | 351.00 | 319.00 | 53.20 | 1480.00 | 1460.00 | 2.73 | T1B3 |
| **mr22c06 06** | 661.00 | 665.00 | 660.00 | 53.20 | 1380.00 | 1390.00 | 0.99 | T1B4 |
| **mr22c07 07** | 364.00 | 386.00 | 389.00 | 53.20 | 1210.00 | 1170.00 | 4.58 | T1B5 |
| **mr22c08 08** | 252.00 | 258.00 | 237.00 | 53.20 | 1350.00 | 1230.00 | 4.03 | T1B6 |
| **mr22c09 09** | 173.00 | 170.00 | 174.00 | 53.20 | 1060.00 | 1060.00 | 0.78 | T1B7 |
| **mr22c10 10** | 164.00 | 160.00 | 158.00 | 53.20 | 1140.00 | 1160.00 | 1.41 | T1B8 |
| **mr22c11 11** | 170.00 | 161.00 | 163.00 | 53.20 | 1090.00 | 1060.00 | 0.82 | T1B9 |
| **mr22c12 12** | 158.00 | 157.00 | 169.00 | 53.20 | 999.00 | 1000.00 | 1.20 | T1B10 |
| **mr22c13 13** | 215.00 | 222.00 | 217.00 | 53.20 | 1180.00 | 1220.00 | 1.15 | T1B11 |
| **mr22c14 14** | 201.00 | 198.00 | 204.00 | 53.20 | 1000.00 | 948.00 | 0.68 | T1B12 |
| **mr22c15 15** | 195.00 | 205.00 | 200.00 | 53.20 | 987.00 | 1020.00 | 1.09 | T1B13 |

**Table S3 (cont.)**

| **Sample #** | **Mg/Ca** | **Sr/Ca** | **Ba/Ca** |
| --- | --- | --- | --- |
|  | **mmol/mol** | **mmol/mol** | **mmol/mol** |
| T1A1 | 0.873240163 | 2.102596623 | 0.000626822 |
| T1A2 | 1.041682427 | 1.746015909 | 0.000702135 |
| T1A3 | 0.496461412 | 1.758311796 | 0.001514102 |
| T1A4 | 0.904269001 | 2.26244315 | 0.001192453 |
| T1A5 | 1.697720722 | 2.496064997 | 0.001161073 |
| T1A6 | 1.555874604 | 2.11489251 | 0.000760189 |
| T1A7 | 1.156932398 | 1.795199456 | 0.000487964 |
| T1A8 | 2.087797546 | 2.139484283 | 0.001200298 |
| T1A9 | 2.987633856 | 2.127188397 | 0.001278749 |
| T1A10 | 2.486739752 | 2.250147263 | 0.003294936 |
| T1A11 | 1.028384354 | 1.85667889 | 0.005530785 |
| T1A12 | 0.540788324 | 1.942750096 | 0.001176763 |
| T1A13 | 0.877672854 | 1.955045983 | 0.001059087 |
| T1B1 | 0.567384471 | 1.524689949 | 0.000363227 |
| T1B2 | 0.802317104 | 1.450914629 | 0.000307527 |
| T1B3 | 1.555874604 | 1.819791229 | 0.002141708 |
| T1B4 | 2.947739635 | 1.696832363 | 0.000776663 |
| T1B5 | 1.711018796 | 1.487802289 | 0.003593049 |
| T1B6 | 1.143634325 | 1.659944703 | 0.003161569 |
| T1B7 | 0.753557501 | 1.303363989 | 0.000611132 |
| T1B8 | 0.709230589 | 1.401731082 | 0.001106157 |
| T1B9 | 0.71366328 | 1.340251649 | 0.000645651 |
| T1B10 | 0.695932515 | 1.22835908 | 0.00094141 |
| T1B11 | 0.984057442 | 1.450914629 | 0.000902185 |
| T1B12 | 0.877672854 | 1.229588669 | 0.000533466 |
| T1B13 | 0.908701692 | 1.213604016 | 0.000855114 |
